# Green Binder Based on Enzymatically Polymerized Eucalypt Kraft Lignin for Fiberboard Manufacturing: A Preliminary Study

**DOI:** 10.3390/polym10060642

**Published:** 2018-06-09

**Authors:** Susana Gouveia, Luis Alberto Otero, Carmen Fernández-Costas, Daniel Filgueira, Ángeles Sanromán, Diego Moldes

**Affiliations:** 1 Department of Chemical Engineering, University of Vigo, Lagoas Marcosende s/n., E-36310 Vigo, Spain; gouveia@uvigo.es (S.G.); mcarmenfc@uvigo.es (C.F.-C.); danmartinez@uvigo.es (D.F.); sanroman@vigo.es (Á.S.); 2 R & D Department of FORESA, Avda, Doña Urraca, 91, Caldas de Reis, 36650 Pontevedra, Spain; l.otero@foresa.com

**Keywords:** wood, *Eucalyptus globulus*, laccase, Kraft lignin, medium-density fiberboards, pilot scale

## Abstract

The capability of laccase from *Myceliophthora thermophila* to drive oxidative polymerization of *Eucalyptus globulus* Kraft lignin (KL) was studied as a previous step before applying this biotechnological approach for the manufacturing of medium-density fiberboards (MDF) at a pilot scale. This method, which improves the self-bonding capacity of wood fibers by lignin enzymatic cross-linking, mimics the natural process of lignification in living plants and trees. An interesting pathway to promote these interactions could be the addition of lignin to the system. The characterization of *E. globulus* KL after enzymatic treatment showed a decrease of phenolic groups as well as the aromatic protons without loss of aromaticity. There was also an extensive oxidative polymerization of the biomolecule. In the manufacture of self-bonded MDF, the synergy generated by the added lignin and laccase provided promising results. Thus, whenever laccase was present in the treatment, MDF showed an increase in mechanical and dimensional stability for increasing amounts of lignin. In a pilot scale, this method produced MDF that meets the requirements of the European standards for both thickness swell (TS) and internal bonding (IB) for indoor applications.

## 1. Introduction

Currently, the wood-based panels industry needs to find natural substitutes of synthetic resins to be a sustainable and eco-friendly industry. The bonding of wood components is achieved when a resinous matrix is formed. The wood boards produced with conventional gluing process show high mechanical strength and relative low swelling when exposed to water.

Up to the present, a great deal of research has been devoted to reducing the environmental impact of wood-based panels industries, more concretely the impact of oil-derived adhesives such as urea-formaldehyde or phenol-formaldehyde. Replacement of fossil phenol by biomass-derived phenols (e.g., lignin, tannins) seems to be a sustainable and environmentally friendly approach [1,2,3]. However, these authors substituted phenol with lignin, but the binders still included formaldehyde, a toxic compound with a significant impact in human health and environment. It is worth noting that the manufacturing of wood-based panels requires the bonding of wood elements, and such unions are due not only to the effect of synthetic resins but also by the intrinsic auto-adhesive properties of wood compounds [4,5]. The auto-adhesive ability suggests that a more environmentally friendly approach to reduce the use of resins is to boost up the inherent capacity of wood elements to bind among themselves.

Most of the studies related to the quest of a binderless fiberboard manufacture process are based on the oxidative modification of lignin [6,7]. The chemical structure of lignin possesses a broad range of chemical groups, i.e., hydroxyl (aliphatic and aromatic), methoxyl, carboxyl or carbonyl, which converts lignin in a raw material with several potential applications [8]. Moreover, such renewable biopolymer is industrially available because it is separated from the polysaccharides during the pulping process of lignocellulosic biomass. Nonetheless, technical lignins, mainly Kraft lignin (KL), are considered by-products and their use is normally limited to energy production in the pulp mills.

The use of phenol-oxidizing enzymes (e.g., laccases, peroxidases) for adhesive applications is based on the capability of enzymes to promote the polymerization and cross-linking of lignin [4,9,10] (Figure S1). The effects of laccases [11,12,13,14] and peroxidases [15,16] over the chemical structure of lignin has been examined by several studies. The authors concluded that both enzymes promote the oxidation of the phenolic groups to phenoxy radicals in a one-electron oxidation. Hence, the incubation of wood fibers with phenol oxidizing enzymes results in the cross-linking of lignin moieties by means of covalent bonds [4,17]. Such mechanism enables the wood fibers adhesion, leading to the manufacturing of wood-based panels with substantial improvements in both mechanical and physical properties. Among the wood composite panels, the manufacturing process for medium-density fiberboards (MDF) has some characteristics which make it particularly suitable for the use of this biotechnology.

The aim of this study is to test the laccase-assisted enzymatic treatment for the manufacturing of binderless *Eucalyptus globulus* fiberboard at pilot scale. Thus, a two-component system manufacturing process adding KL to wood fibers was tested and the effect of incubation time as well as the amount of lignin added to the board was studied. The effect on the physical and mechanical properties of the resulting wood boards was assessed. To a better understanding of the enzymatic reactions involving lignin during fiber enzymatic treatments, isolated KL was subjected to the same treatment and comparative studies of the lignins, before and after enzymatic action, were performed by several analytical techniques (FTIR, HPLC-SEC, 2D NMR).

The MDF manufacturing process tested in this study is presented as an interesting lignin valorization in the context of circular economy with the additional environmental benefits of removing toxic binding materials from the conventional manufacturing process.

## 2. Materials and Methods

### 2.1. Lignin

Black liquor from the Kraft cooking process of *Eucalyptus globulus* was supplied by ENCE (Pontevedra, Spain). Kraft lignin (KL) was obtained by acidic precipitation of the black liquor as follows. Black liquor was diluted in water (1:1) and the pH was lowered to 2.5 by addition of sulfuric acid 4 M. The solution was left under stirring for 30 min. The precipitate was centrifuged, and the isolated KL was washed twice with acidified water (pH 2.5). KL was oven-dried overnight at 60 °C, and then it was milled in an agate mortar and stored in amber glass bottle until use.

### 2.2. Laccase Enzyme

Laccase from *Myceliophthora thermophila* (NS51003) was kindly supplied by Novozymes (Bagsvaerd, Denmark). Commercial laccase was desalted using a PD-10 desalting column with Sephadex G-25 Medium (General Electric, Norwalk, CT, USA) following the supplier recommended protocol.

Laccase activity was determined spectrophotometrically by oxidation of 2,2′-azino-*bis* (3-ethylbenzothiazoline-6-sulphonic acid) (ABTS) at 436 nm (ε = 29300 M^−1^cm^−1^) in potassium phosphate buffer pH 7.3 at 25 °C. One activity unit (U) was defined as the amount of enzyme that oxidized 1 μmol of substrate per min.

### 2.3. Wood Fibers

*Eucalyptus globulus* fibers were supplied by the MDF board plant of FINSA industries (Padrón, Spain). The fibers were dried in a flash drier until a moisture content of 2%.

### 2.4. KL Enzymatic Polymerization

Lignin was solubilized in phosphate buffer pH 7.3 (100 mM) obtaining a solution of 1.5 g·L^−1^. The desalted laccase was added to 90 mL of the latter solution to reach a final activity of 2 U·mL^−1^. The reaction took place at 70 °C for 2 h in an orbital shaker. The reaction was stopped by lowering the pH to 2.0 causing enzyme deactivation and lignin precipitation. The reaction product was filtered and washed twice with acidified water (pH 2.5) and oven-dried at 60 °C overnight.

### 2.5. KL Characterization

#### 2.5.1. Determination of Phenolic Content

Phenolic content was evaluated as described by [18]. Initially, KL solutions of 0.5 g·L^−1^ in NaOH 0.05 M were prepared. Then, 1 mL of KL solution, 3 mL of Folin and Ciocalteu reagent and 30 mL of distilled water were added into a volumetric flask and mixed thoroughly. After 5–8 min of stirring, 10 mL of 20% (*w*/*w*) sodium carbonate solution was added and the volume was adjusted to 50 mL with DI (deionized) water. The mixture was stirred for 2 h and finally, the absorbance at 760 nm was measured. The phenolic content test was run in duplicate. Vanillin standard solutions (0–5 mM) were used for calibration.

#### 2.5.2. Fourier Transform Infrared Spectroscopy (FTIR)

Solid KL samples were placed to dry for 10 min under an infrared lamp. The FTIR spectra were recorded with a Jasco FT/IR-4100 (Easton, PA, USA), equipped with attenuated total reflectance (ATR) in absorbance mode using a frequency range of 650–4000 cm^−1^. Each spectrum, which accumulated 32 scans at 4 cm^−1^ resolution, was then analyzed with the OMNIC 32 software.

The absorption bands were assigned as suggested by [19]. The spectrum was baselined and ATR corrected and the bands intensities were normalized referring aromatic skeletal vibration (around 1510 cm^−1^).

#### 2.5.3. Molecular Weight Distribution

Lignin samples were dissolved in NaOH 0.05 M (final concentration of 0.5 g·L^−1^), and left in agitation for 8 h at 300 rpm and 40 °C. Once totally dissolved, the samples were filtrated with PVDF 0.2 μm syringe filter.

Size-exclusion chromatography was performed in a Jasco HPLC system (Easton, PA, USA) (AS 1555 auto sampler; PU 2080 plus pump, UV 975 detector) equipped with two GPC columns (Phenomenex, Torrance, CA, USA) coupled in series (GPC P4000 and P5000, both 300 mm × 7.8 mm) and a safeguard column (35 mm × 7.8mm). The injection volume was 100 μL, and the isocratic flow (NaOH 0.05 M) was pumped at a rate of 1 mL·min^−1^ at 25 °C for 26 min. Detection was performed with a UV detector at 254 nm. Data was recorded and analyzed with ChromNAV GPC software. Calibration curve was obtained with polystyrene polymer standards (Phenomenex) with molecular weights of 891, 1670, 6430, 10,200, 33,500, 65,400, 158,000, 305,000, 976,000 and 2,350,000 Da. Concentrations and injection volumes were performed according to the manufacturer’s specifications. The mobile phase was the same used as solvent for KL samples, i.e., 0.05 M NaOH.

#### 2.5.4. Nuclear Magnetic Resonance (NMR)

NMR techniques, namely proton nuclear magnetic resonance spectroscopy (^1^H), carbon nuclear magnetic resonance spectroscopy (^13^C) and Heteronuclear Single Quantum Coherence (HSQC) were used for KL characterization. KL samples were previously acetylated to enhance their solubility in the NMR solvent. An adaptation of the method described by [20] was performed to acetylate the samples. Approximately 100 mg of KL was placed in a dry acetylation vial and 500 μL of pyridine were added. The vial was sealed and constantly stirred at 37 °C during 1 h or until the KL full sample dissolution. Afterwards, 1 mL of acetyl anhydride was added and left reacting with constant agitation at 37 °C for 48 h. Finally, once the acetylation process was over, 440 μL of methanol were added to remove the excess of pyridine and acetyl anhydride. After 2 h of stirring, the content of the vial was dried and milled with and agate mortar and pestle.

All spectra have been recorded at 25 °C on a Bruker Avance 400 MHz (Billerica, MA, USA), equipped with a z-gradient 5-mm QNP probe. Chemical shifts were referred to the solvent signals, δ(^1^H) = 2.5 ppm, δ(^13^C) = 39.5 ppm.

##### ^1^H NMR

50 mg of both acetylated and non-acetylated KL were dissolved in 750 μL of deuterated dimethyl sulfoxide (DMSO-*d*6). The relaxation delay was 1 s and a flip angle of 30° was used. The number of scans was 16 with an acquisition time of 6.3 s. Spectra were processed using an exponential weighting function of 0.3 Hz prior to Fourier transformation.

##### ^13^C NMR

50 mg of KL were dissolved in 750 μL of DMSO-*d*6. The relaxation delay was 5 s, and a flip angle of 30° was used. The number of scans was 50,000 with an acquisition time of 1.4 s.

##### HSQC

KL samples (150 mg) were dissolved in 750 μL of DMSO-*d*6. The number of complex points collected was 2048 for the ^1^H dimension and a recycle delay of 5.28 s (5 s relaxation delay and 0.28 s acquisition time) was chosen. The number of transients was 128 and 256 time increments were recorded in the ^13^C dimension.

### 2.6. Medium-Density Fiberboards Production

#### 2.6.1. Enzymatic Fiber Pre-Treatments

*E. globulus* fibers were submitted to different treatments as listed in Table 1. Regarding to one-component system, laccase was air-pressurized sprayed to the dried fibers on a rotary drum blender until homogenization. However, in the case of two-component system (fiber and KL), KL was premixed with the fibers in the rotary blender prior to enzyme addition. Enzyme was added as commercially supplied and no pH adjustment was carried out. After homogenization, to allow sufficient time for the enzymatic reactions take place, fibers (or fibers and added KL) were left in an oven at 70 °C for 2 h, in a vessel without agitation. The vessel was covered with aluminum foil to avoid water evaporation, except when initial moisture was 20% or higher; in such cases the vessel was uncovered, and the moisture controlled every 30 min to ensure minimum moisture of 10–12%.

#### 2.6.2. MDF Preparation

After the enzymatic treatment, fibers were pressed in a mold, producing an approximately cubic mat (≈250 mm × 250 mm × 250 mm). The resulting mat was cold pressed for about 2 min to an approximate height of 40 mm. The mat was taken in hot press (press platens were maintained at 200°C) and reduced to its final thickness in a dynamic three step cycle with a total time of 8 min. In step one, the mat was pressed to 22 mm for 232 s and, in the second and third step, the board was pressed to a height of 18.5 mm and 16.7 mm respectively, both for 124 s.

**Figure 1:**
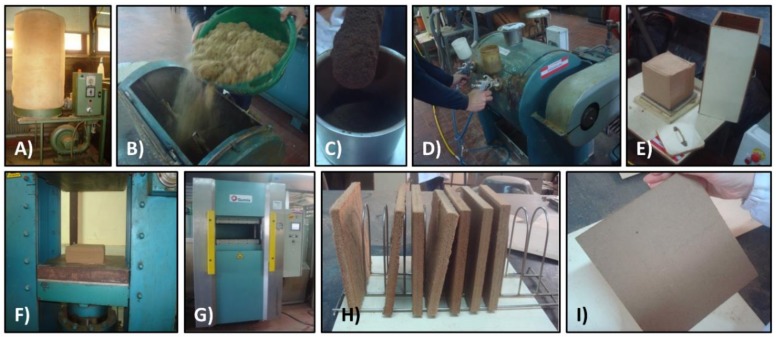
Binderless MDF pilot scale manufacturing procedure: (**A**) Drying of eucalyptus fibers; (**B**) Addition of fibers with 2% moisture to drum blender; (**C**) *E. globulus* KL addition; (**D**) Laccase spraying with air pressure; (**E**) Mat manually pressed; (**F**) Mat cold pressing; (**G**) 3 steps hot pressing cycle; (**H**,**I**) Final MDF boards.

The complete manufacturing process is described in Figure 1, and the process parameters are summarized in Table 1.

#### 2.6.3. Medium-Density Fiberboard Properties

The internal bond strength (IB) was determined according to EN 319 [21]. Thickness swell (TS) and water absorption (WA) were determined using specimens of (50 ± 1) mm × (50 ± 1) mm. After an aging cycle where the specimens were submersed for 24 h in an upright position in water at 20 °C, the excess of water was drained. Specimen’s thickness and mass was measured prior and after the immersion.

## 3. Results and Discussion

### 3.1. Kraft Lignin Enzymatic Polymerization and Characterization

Laccase enzyme is one of the most inexpensive enzymes widely available in the market. Commercial laccase from *M. thermophila* showed the ability to oxidize and polymerize Kraft lignin (KL). The extension of this polymerization depended on several parameters such as lignin origin, pH, temperature, phenolic mediators or reaction time [11,12,22,23]. The aim of this study was to test the capability of the enzymatic treatment of KL in the manufacturing of MDF with no synthetic resins. The enzymatic treatment was tested to enhance the cross-linking among lignin molecules to produce stable and durable MDF from *E. globulus* fibers and KL. The experimental conditions used in these studies were those previously identified to favor the enzymatic polymerization of *E. globulus* KL [11].

**Figure 2:**
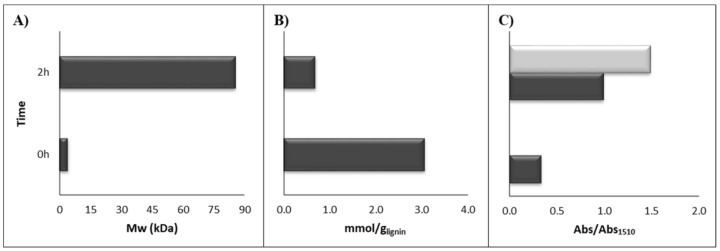
Comparison between untreated (0 h) and enzymatically treated (2 h) *E. globulus* KL: (**A**) Molecular weight; (**B**) Phenolic content; (**C**) Conjugated C=O FTIR absorbance referenced to aromatic skeletal vibration (light gray) and non-conjugated C=O FTIR absorbance referenced to aromatic skeletal vibration (dark gray).

The oxidative enzymatic treatment of KL with laccase induced clear changes in the structure of lignin as it was observed in the reaction vessel during the reaction. Hence, the resulting enzymatically-treated lignin showed a much darker brown color compared to the original lignin. Moreover, the polymerized KL required a very long stirring time (1 day) to be dissolved in NaOH 0.05 M whereas the original KL was easily dissolved in just a few seconds. Gel permeation chromatography, phenolic content, FTIR and NMR (^1^H, ^13^C and HSQC) analyses were used to characterize the enzymatically-polymerized KL. Detailed analytical procedures, results and discussion can be found in previous publications [11,12,24]. Gel permeation chromatography permits to compare the average molecular weight of the original and polymerized KL (Figure S2). As observed in Figure 2A, the enzymatically-treated KL showed a strong increase in the average molecular weight (17-fold) compared to the original KL. At the same time, the enzymatic polymerization was associated with a loss of the lignin’s phenolic content (Figure 2B). Such results evidenced that laccase oxidized the KL’s phenolic moieties to phenoxy radicals, which were coupled among themselves leading to a remarkable increase in the molecular weight and a lower phenolic content of the KL. The FTIR spectra analyses of both polymerized and original KL detected that polymerized KL showed a higher signal of the peaks associated with C=O bonds, for both non-conjugated (1718–1703 cm^−1^) and conjugated C=O bonds (1655–1654 cm^−1^) (Figure S3). It is important to stress that the conjugated C=O were not detected in the original KL (Figure 2C).

**Figure 3:**
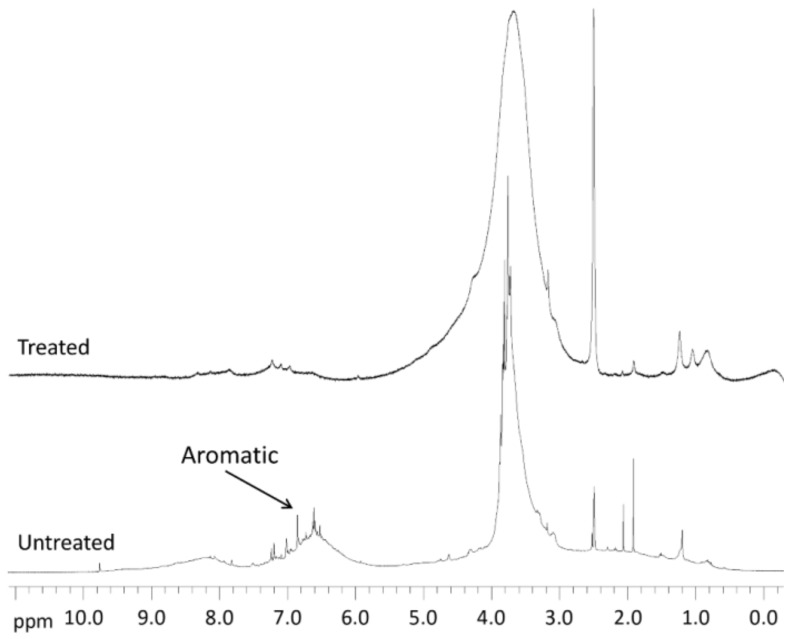
^1^H NMR spectra of non-acetylated *E. globulus* Kraft lignin samples.

NMR techniques, particularly 2D-NMR such as HSQC, are powerful tools in the identification and quantification of lignin main structures [25,26]. ^1^H NMR analysis of non-acetylated KL (Figure 3) indicates a pronounced decrease of the aromatic protons in KL after laccase-mediated polymerization. The comparison of ^1^H NMR spectra, before and after polymerization, shows a wide band associated to methoxyl groups (4.2–3.1 ppm) after enzymatic treatment whereas most of the aromatic protons disappeared (7.0–6.0 ppm). In addition, benzaldehyde protons, with a chemical shift of 9.78 ppm, also disappeared after the polymerized KL. The disappearance of aromatic proton signals was not totally unexpected as it has been previously reported with lignosulfonates by [27].

**Figure 4:**
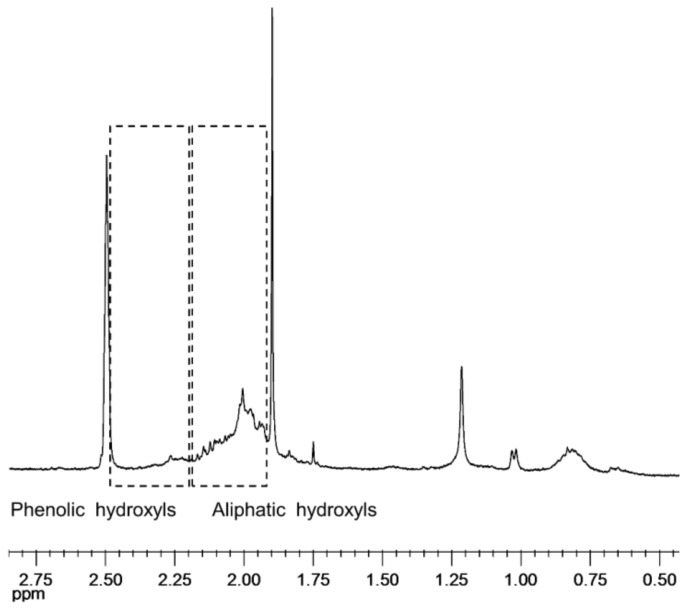
Expanded region of ^1^H NMR spectrum of an enzymatically treated *E. globulus* KL subjected to acetylation.

Sample acetylation improved the KL solubilization and allows differentiating the aliphatic from the aromatic hydroxyl groups. However, the process of acetylation caused structural changes and the spectra obtained for the acetylated samples showed poorer signals. Nevertheless, ^1^H NMR analysis of acetylated samples (Figure 4) evidenced that nearly all aromatic hydroxyls have been attacked by laccase while some aliphatic hydroxyls remained in the polymerized KL.

**Figure 5:**
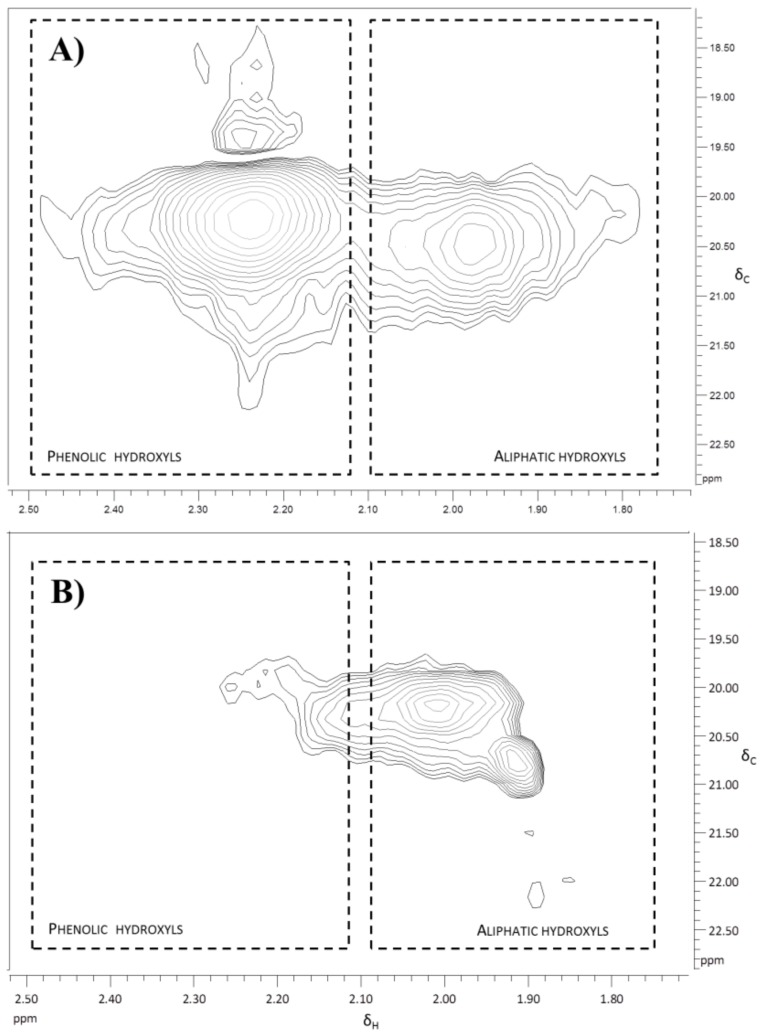
Hydroxyl expanded region of HSQC spectrum of acetylated *E. globulus* KL: (**A**) Untreated; (**B**) After enzymatic polymerization.

This result was even clearer in the HSQC spectrum (Figure 5), where there was no correlation for aromatic hydroxyls and there was only a small area corresponding to the aliphatic hydroxyls.

**Figure 6:**
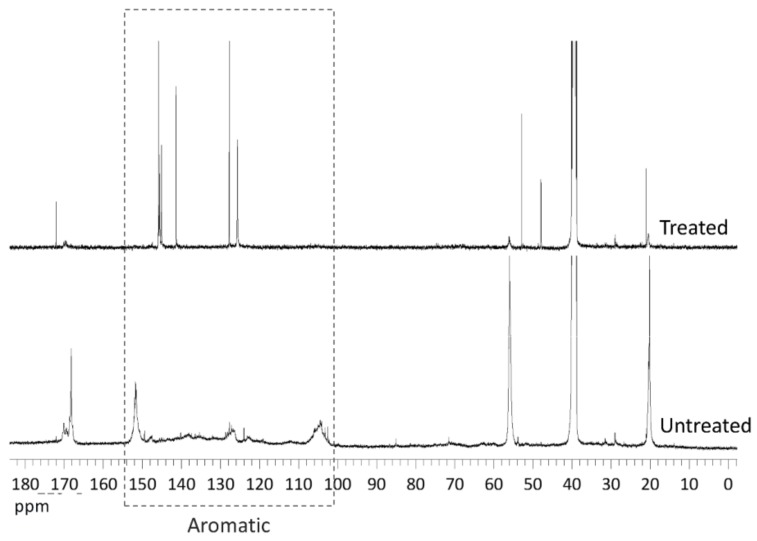
^13^C NMR spectra of acetylated *E. globulus* KL.

^13^C NMR also revealed significant differences between KL samples (Figure 6). After the enzymatic treatment, a remarkable increase in the signal at 172.0 ppm (carboxyl groups) was accompanied by the disappearance of the signal corresponding to phenolic hydroxyls (168.1 ppm), whereas the weak signals of primary and secondary aliphatic hydroxyls remained almost unchanged (170.0 ppm and 169.4 ppm, respectively). On the other hand, after the enzymatic treatment, strong signals of aromatic carbons (160–100 ppm) remained in ^13^C NMR spectrum proving that benzene rings were not affected in the polymerization. This suggests that the polymerization observed by size-exclusion chromatography could be explained by the laccase mode of action: the enzyme initiated the oxidation of phenolic groups into stabilized radicals that subsequently, undergone radical-radical coupling through phenyl ether-carbon and carbon-carbon links. These new bonds yield to the observed increase in molecular weight without destruction of the aromatic KL backbone. Similar behavior was observed by other authors in the polymerization of commercial lignosulphonates and lignin models by laccase [27,28].

### 3.2. MDF Manufacture

Manufacturing of binderless MDF with an enzymatic pre-treatment by dry process were performed under the conditions specified in Table 1. The main objective of these tests was to assess the possible MDF manufacture with only an enzymatic treatment of the wood fibers and/or the addition of KL. The experimental conditions in the MDF manufacturing, such as the amount of enzyme and KL added were adjusted to improve the fiberboard characteristics, maximizing its internal bonding (IB) and minimizing the water absorption and thickness swelling (TS). The dynamic pressing cycle was not optimized in the present study. The temperature and press factor (s·mm^−1^ board thickness) used during hot pressing were the same used by the industrial partner when testing, in pilot plant, the production of MDF with synthetic resins. These conditions would have to be improved in an industrial scale, since the typical values in the industrial process are around 10 s pressing time per mm board thickness.

Nowadays, there are two main industrial processes to obtain high quality wood fibers for MDF manufacturing: thermal and thermo-mechanical pulping process. In both processes, high temperatures and pressure can efficiently defibrate wood, producing a raw material with suitable properties for fiberboard manufacturing [29]. Importantly, the heat associated with high pressures causes chemical changes in the fibers. On one hand, small fragments of lignin are formed in the defibration process, that finally settle in the surface of the fibers. These fragments show a high reactivity due to the presence of phenolic radicals in their structure [30,31]. On the other hand, a glassy layer of hardened lignin is formed on the surface of the fiber because defibration takes place at temperatures above lignin’s glass transition temperature [32]. This glassy crust on the fiber surface is a barrier for the enzymatic action in wood fiber’s lignin. However, when enzymes are used in the manufacturing process, the initially existing crust is loosened, and lignin is again available for laccase oxidative action. Furthermore, the results found in literature for MDF production, at laboratory and pilot-scale (Table 2), are revealing that laccases can oxidize and polymerize in an efficient way not only isolated lignins, but also lignins in a fiber-bound state. These results indicate that the glassy form of lignin, which covers the wood fibers after defibration, is not able to inhibit, at least completely, the oxidative action of laccase over lignin.

Table 3 summarizes the properties of the binderless MDF manufactured under various experimental conditions. The enzyme dosage was kept constant throughout the tests (29 U/g of dry fiber). This value was selected considering our previous studies regarding lignin polymerization [11] and the information available in the literature (Table 2). A control experiment (T1) was performed by adding to the fiber nothing but the necessary water for proper heat conduction, i.e., 10–13% moisture content. These conditions were set to ensure that the temperature in the center of the board exceeds the lignin glass transition temperature because lignin plasticization have been considered partially responsible for board adhesion [37].

#### 3.2.1. One-Component System

According to the results shown in Table 3, the fiber pre-treatment with laccase (T2) did not improve the MDF properties when compared to the untreated control (T1). The increase of the enzymatic treatment time, until 24 h (T3), did not show any improvement in the IB of the MDF. Furthermore, no dimensional stability was showed by the enzymatically-treated MDFs (both T2 and T3), which completely detached when exposed to water for 24 h. The poor results indicate an issue in the manufacturing conditions.

Among the few publications using dry incubation process for one-component binderless MDF production (Table 2), [9] were able to produce fiberboards with reasonably good characteristics. However, there are some noticeable differences between this study and [9]. One is the fiber source: lignins from different wood species have distinct chemical and physical characteristics, such as syringyl/guaiacyl ratio, phenolic content, molecular weight distribution, etc., that may considerably affect the enzymatic polymerization [11,38,39]. Also, the thickness and density of the MDF were very different in both studies, and this may have an important impact on the final MDF properties [40]. In a more recent work [36], Euring et al. were able to achieve MDF with good mechanical properties with an IB and modulus of rupture (MOR) of 0.42 and 20 Mpa respectively, yet, a TS of 50% was still an issue. To improve the dimensional stability of the fiberboards, the authors tested a laccase-mediator-system. The best results were obtained using 4-hydroxybenzoic acid as mediator, leading to a TS of 19% and a simultaneous improvement in the mechanical properties. Other authors [35] used a laccase-mediator-system to introduce a water-based wax as hydrophobic agent in the manufacturing of a binderless MDF. Until then, the combined use of hydrophobic agents and enzymes were supposed to be incompatible because the hydrophobic agents coated the fibers hindering the enzymatic access to lignin [4]. However, [35] obtained an MDF with IB as high as 0.9 MPa and TS of 17% using 1% of hydrowax in a laccase treatment with vanillic alcohol as mediator.

Other alternative to enhance the MDF properties could be the addition of technical lignin to the fibers before the enzymatic treatment. This is the so-called two-component system. Technical lignins are those obtained after the lignocellulosic pulping process (e.g., KL). These lignins have a molecular structure considerably different with respect to native lignin. Moreover, technical lignins will have distinct characteristics in composition and structure depending on the pulping process.

Among the several pulping processes to fragment lignocellulose into their major compounds, hemicellulose, cellulose and lignin, Kraft process is the predominant. During Kraft digestion, depolymerization of native lignin mainly occurs through the extensive cleavage of β-O-4′ ether bonds. The resulting KL has not only a higher amount of phenolic hydroxyl groups but also, biphenyl and other condensed structures are less formed than in other pulping processes [41]. As the main goal during the Kraft process is to achieve high quality cellulose, the large amount of KL obtained is considered a by-product.

In modern Kraft mills, on-site Kraft lignin burning for steam and energy production ensures not only the energy self-sufficiency of these industrial units but also an energetic surplus. However, the incineration (even with energy recovery) is considered the last option for any by-product valorization. As many of the major Kraft pulp producers are considering the conversion of their units in biorefineries, the valorization of Kraft lignin, which is produced in huge quantities, is crucial. Nowadays, Kraft powder lignin is already widely available in the market for various proposes other than incineration.

Therefore, the high availability and high phenolic content of KL, converts such by-product of the pulp industry in a suitable substrate to be used in laccase-assisted processes such as the manufacturing of eco-friendly binderless MDF.

#### 3.2.2. Two-Component System

The two-component system, consisting on the addition of *E. globulus* KL and laccase to the fibers, was tested for the manufacturing of MDF at pilot-scale. It was expected that the enzymatic treatment enhanced the copolymerization between the added KL and the lignin on the wood fibers. Taking into account that solid *E. globulus* KL was added to the mixture of wood fibers and enzyme, the enzyme dosage was adjusted (T4–T9) as shown in Table 3. Hence, the enzymatic dosage used is no longer referred to fiber weight but to the amount of added KL (190 U of laccase per g of KL).

In the case of fiberboards composed only by wood fibers and KL (T4), with no enzyme addition, the properties obtained were similar to the control fiberboards (T1), suggesting the non-significant effect of single KL addition. When laccase was sprayed in the mixture of wood fibers jointly with KL (T5), the cohesion of the board was improved. Such small improvement (IB was 0.06 MPa) suggests that the laccase-KL system was able to form cross-linking between the wood fiber’s lignin and the added KL. However, the water absorption and TS of the MDF were not measurable because the board detached before the end of the analysis.

Previous studies have combined the addition of lignin, or lignin-like phenolic compounds, in a two-component system binder for MDF production [36]. The phenolic groups offered by the added KL are more accessible to enzymatic action than those found within the wood fibers [42]. Thus, the advantage of a two-component system is not limited to provide a larger number of phenolic groups, but also ensure that those phenolic groups are readily available for enzymatic action, enabling the formation of a greater number of phenoxy radicals. In fact, [36], improved the IB of MDF with the addition of lignosulphonates to the enzymatic treatment of wood fibers. Nonetheless, the TS values obtained were still worse than those obtained with urea-formaldehyde resin. Probably, the hydrophilic nature of lignosulphonates led to less favorable results of MDF dimensional stability. Nonetheless, due to its higher hydrophobic character, KL could be an interesting alternative to lignosulfonates for the manufacturing of binderless MDF in a two components system.

The effect of the KL amount added to the manufacturing process of MDF was also assessed, but keeping constant the ratio of enzyme activity per g of added KL. The increase of KL amount added to the enzymatic treatment (T7) of the wood fibers had a remarkable effect. IB was clearly improved (5-fold), indicating that a higher interlinking between both the wood fiber‘s lignin and the KL was achieved. Moreover, the water absorption and the TS were significantly improved respect to previous treatments. Nevertheless, the amount of polymerized KL was not enough to give an optimal protection against water. It is worth noticing that T6 and T7 had the same amount of KL but, laccase was not present in T6. Therefore, there was no enzymatic induced cross-linking between the KL and the lignin moieties in the wood fibers. Furthermore, the decrease in the lignin phenolic groups (which are the hydrophilic groups of lignin) caused by laccase oxidation (Figure 2B, Figure 4 and Figure 6), could considerably contribute to the lower water absorption and TS observed in the MDF of T7.

**Figure 7:**
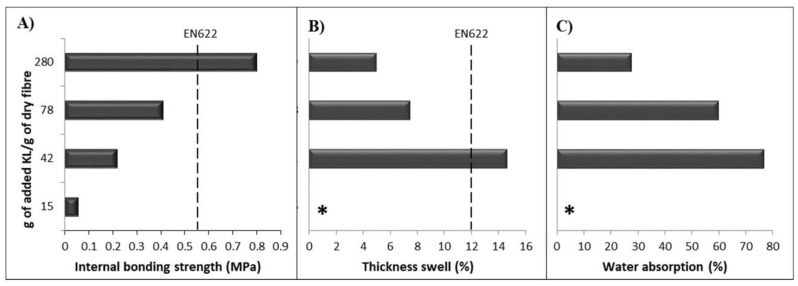
Effect of *E. globulus* KL addition in MDF properties. In all cases 190 U laccase·g^−1^ of KL was used. (**A**) Internal bond; (**B**) Thickness swell after 24 h immersion cycle; (**C**) Water absorption after 24 h immersion cycle. * Indicates MDF detachment when submersed.

Given these results, in T8 and T9, the amount of KL was increased even further. As a result, a clear improvement of board properties was obtained, enabling the manufacturing of MDF with both IB and dimensional stability (Figure 7). Moreover, the MDF properties from test T9 were even better than those reported by [36] in a lignin-laccase-mediator-system. It is noticeable that the T9 MDF (KL + laccase with no mediators) showed similar IB but much better TS than [36] results. As it was commented before, the different industrial lignin used in these studies could be the main reason for the different TS results.

Importantly, T8 and T9 binderless MDFs reached the standards required for indoor application. Such standards depend on the target market, for instance, the American norm for general interior uses, ANSI A.208.2-2002 [43], requires a minimum IB of 0.30 MPa and a TS no higher than 10% for an MDF Grade 110. The equivalent European standard EN 622-5:2010 [44] is more demanding for IB requirements (≥0.55 MPa) but more tolerant for the TS limit (≤12%). Thus, T9 MDF manufactured in the present study met both the European and American standards for dry environments in both studied properties (IB and TS). This set of tests have proved that it is possible the manufacturing of fiberboards with no synthetic resins. In our tests, the binding among the wood fibers may be attained by the plasticizing effect of the lignin (added lignin and the lignin in the fibers) when heated, and the cross-linking catalyzed by the laccase enzyme. The plasticizing effect of lignin was observed in the tests T4 and T6 with no enzyme. The combination of enzyme and the plasticizing effect of lignin could result in a synergistic effect giving the fiberboard in test T9 the adequate properties to be marketed.

It is noteworthy that three quarters of T9 MDF weight is composed of *E. globulus* KL, so it could be considered a lignin-based matrix with wood fibers working as a reinforcing material. Thus, the proposed binderless process of MDF production, in addition to the environmental benefits resulting from the removal of synthetic resins, is a promising pathway of KL valorization, a waste stream of the pulp and paper industry.

## 4. Conclusions

The oxidation caused by laccase from *M. thermophila* led to a strong polymerization of an isolated industrial KL from *E. globulus*. Although a significant loss of hydroxyls and aromatic protons was detected by NMR, there was no disruption on the aromatic backbone of the polymer. The use of KL with laccase in a two-component system enabled the manufacturing of MDFs totally free of synthetic resins or additives, with *E. globulus* as the main raw material. Laccase catalyzed the cross-linking between different lignin molecules forming a 3D structure that confers to the MDF dimension stability, hydrophobicity, and mechanical resistance. The MDFs obtained with laccase + KL (two components system) showed remarkable high internal bonding and low thickness swelling. The boards met the European and American standards for indoor applications. These features and the high availability of KL can make the laccase-assisted polymerization of KL as a fully green strategy to substitute synthetic adhesives in the wood-panels industry.

## Figures and Tables

**Table 1:** Experimental conditions and fiberboards composition.

Treatment	*One-Component System*			*Two-Component System*					
	T1	T2	T3	T4	T5	T6	T7	T8	T9
Dry fiber (g)	520	520	520	520	520	421	421	325	145
KL (% *w*/*w* dry fiber)	0	0	0	15	15	42	42	78	280
Water added (% *w*/*w* dry fiber)	12			12		12			
Enzyme dosage (U·g^−1^ dry fiber)	0	29	29						
Enzyme dosage (U·g^−1^ KL)				0	190	0	190	190	190
Moisture content after blending (%)	12	12	12	11	11	11	20	26	36
Incubation time at 70°C (h)		2	24		2		2	2	2
Moisture content before pressing (%)	11	11	11	10	10	10	12	13	15
Fixed experimental conditions									
Wood fiber origin	*Eucalyptus globulus* (100%)								
KL origin	*Eucalyptus globulus* (100%)								
Target board density	650–700 kg·m^−3^								
Cold pressing time	120 s								
Cold press plate position (H)	30 mm								
Hot pressing temperature	200 °C								
Hot press plate position (H)	22 mm		18.5 mm		16.7 mm				
Hot pressing times	232 s		124 s		124 s				
Final board size (W × L × H)	250 mm × 250 mm × 16.7 mm								

**Table 2:** Examples found in literature of binderless MDF glued by auto-adhesion of treated fibers.

Fiber	Incubation Conditions						Pressing Conditions		Scale	Board Properties						Reference
		Laccase	T (°C)	Time (h)	pH	Enzyme Dosage (U·g^−1^ Fiber)	Press Factor (s/mm)	T (°C)		Density (kg·m^−3^)	MOE (GPa)	MOR (MPa)	IB (MPa)	WA (%)	TS (%)	
*Fagus sylvatica*	Control	Heat deactivated	20	1.0	5	0	100	200	Lab.	850	3.42	25.3	0.91	143	45	[9]
	Treated	*Trametes versicolor*				3.5				895	4.02	41.7	1.57	72	19	
*Fagus sylvatica*	Control	Untreated	50	0.5	7	0	25	200	Pilot	820	(b)	(b)	0.33	224	146	[4]
	Treated	*M. thermophila*				6				858	3.70	40.1	0.82	109	69	
						24				868	3.95	46.0	0.93	92	46	
Leaf sheath from commercial plants	Control	Untreated	30	1.0	6	0	160	200	Lab.	1100	1.3	13.3	-	268.3	218.8	[6]
	Treated	*Aspergillus oryzae*				6				1100	13.3	17.5	-	82.4	67.5	
						12				1100	268.3	18.6	-	79.7	31.4	
						24				1100	218.8	18.7	-	80.5	30.1	
*P. sylvestris (90%)* *P. radiata (10%)*	Control	Heat deactivated	≤120	≈0.5	6	0	22	200	Pilot	800	-	≈10	≈0.1	-	≈122	[33]
	Treated	*Trametes villosa*				100				800	-	≈20	≈0.38	-	≈62	
*Havea brasiliensis*	Control	-	25	1.0	5	-	40	200	Lab.	-	-	-	-	-	-	[34]
	Treated	*Trametes villosa*				9				750	3.6	9.3	0.67	-	-	
*Spruce. (80%)* *Fir (20%)*	Control	Buffer + wax	≤165	≈0.5	6	100	60	200	Pilot	850	-	19	<0.1	-	32	[35]
	Treated	*Trametes villosa* + wax				3.5				850	-	37	0.32	-	20	
*Spruce. (80%)* *Fir (20%)*	Control	Buffer	-	-	6	100	12	190	Pilot	750	-	12	<0.1	-	100	[36]
	Treated	*Trametes villosa*									-	20	0.42	-	50	

(b) delaminated boards; MOE: modulus of elasticity: MOR: modulus of rupture; IB: internal bonding; WA: water absorption; TS: thickness swell.

**Table 3:** Summary of MDF manufacturing conditions and MDF properties.

	Treatment								
	*One-Component System*			*Two-Component System*					
	T1	T2	T3	T4	T5	T6	T7	T8	T9
**Treatment**									
KL (% *w*/*w* dry fiber)	0	0	0	15	15	42	42	78	280
Enzyme dose (U·g^−1^fiber)	0	29	29						
Enzyme dose (U·g^−1^ KL)				0	190	0	190	190	190
Incubation time (h)		2	24		2		2	2	2
**Board properties**									
Density (kg·m^−3^)	661	670	633	697	705	698	688	785	831
IB (MPa)	<0.01	<0.01	<0.01	<0.01	0.06	0.04	0.22	0.41	0.80
WA (%)	D	D	D	D	D	134.7	76.8	60.0	27.8
TS (%)	D	D	D	D	D	55.2	14.6	7.5	5.0

D—detached.

## Supplementary Materials

The following are available online at http://www.mdpi.com/2073-4360/10/6/642/s1, Figure S1: Representation of laccase redox cycle with: A) phenolic substrate; B) phenolic and non-phenolic substrate with a mediator title, Figure S2: Size exclusion chromatogram of untreated KL (blue line) and enzymatically treated KL (Black line), Figure S3: FTIR spectra of untreated KL (blue line) and enzymatically treated KL (Black line).
