# Supplementary figures and images for: Green Binder Based on Enzymatically Polymerized Eucalypt Kraft Lignin for Fiberboard Manufacturing: A Preliminary Study
Source: Polymers (Basel). 2018 Jun 9;10(6):642. doi: 10.3390/polym10060642 (PMC6403738; doi:10.3390/polym10060642)

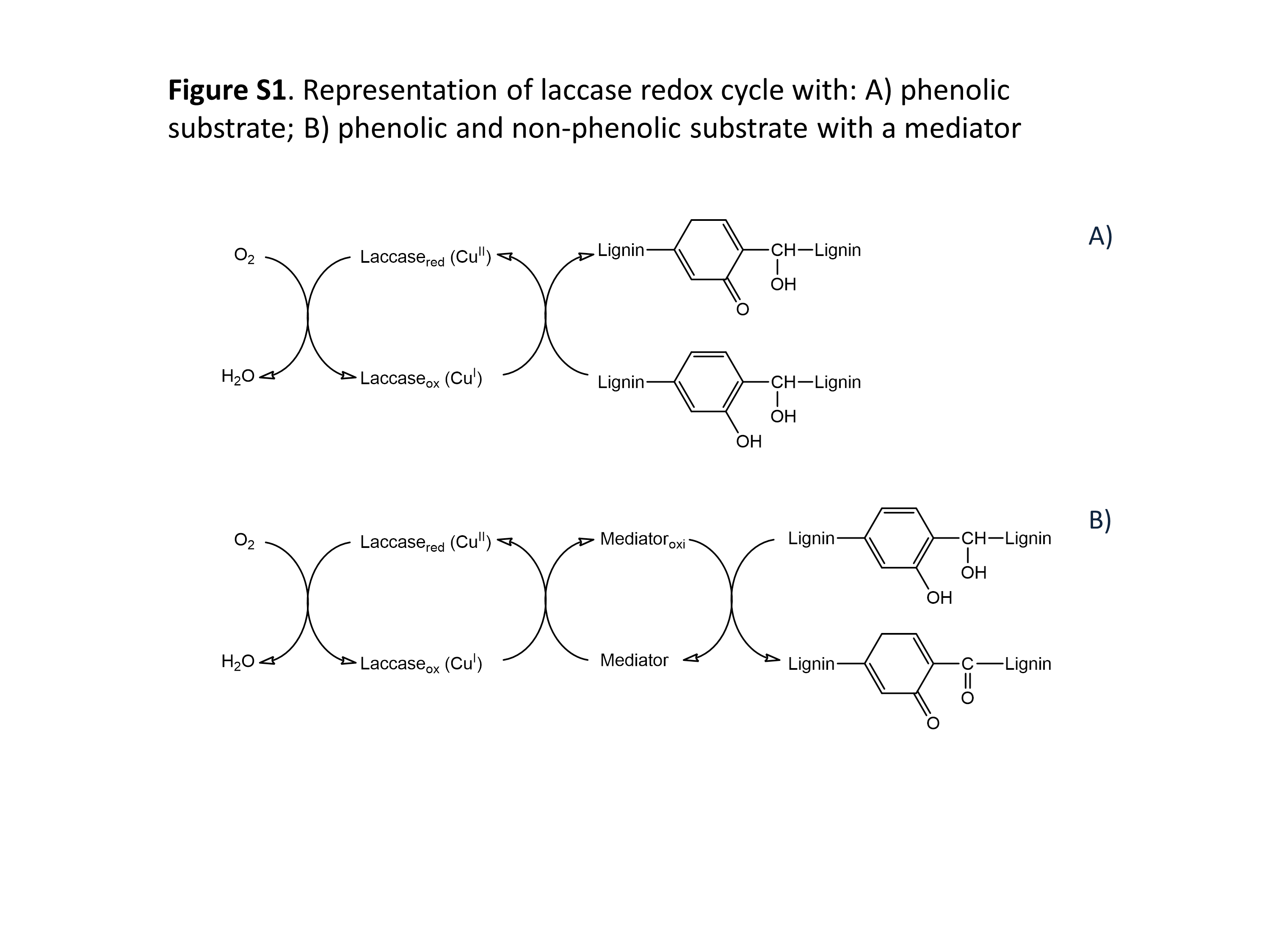

Supplement: Supplementary file 1 [file polymers-10-00642-s001.zip › Figure S1.TIF]

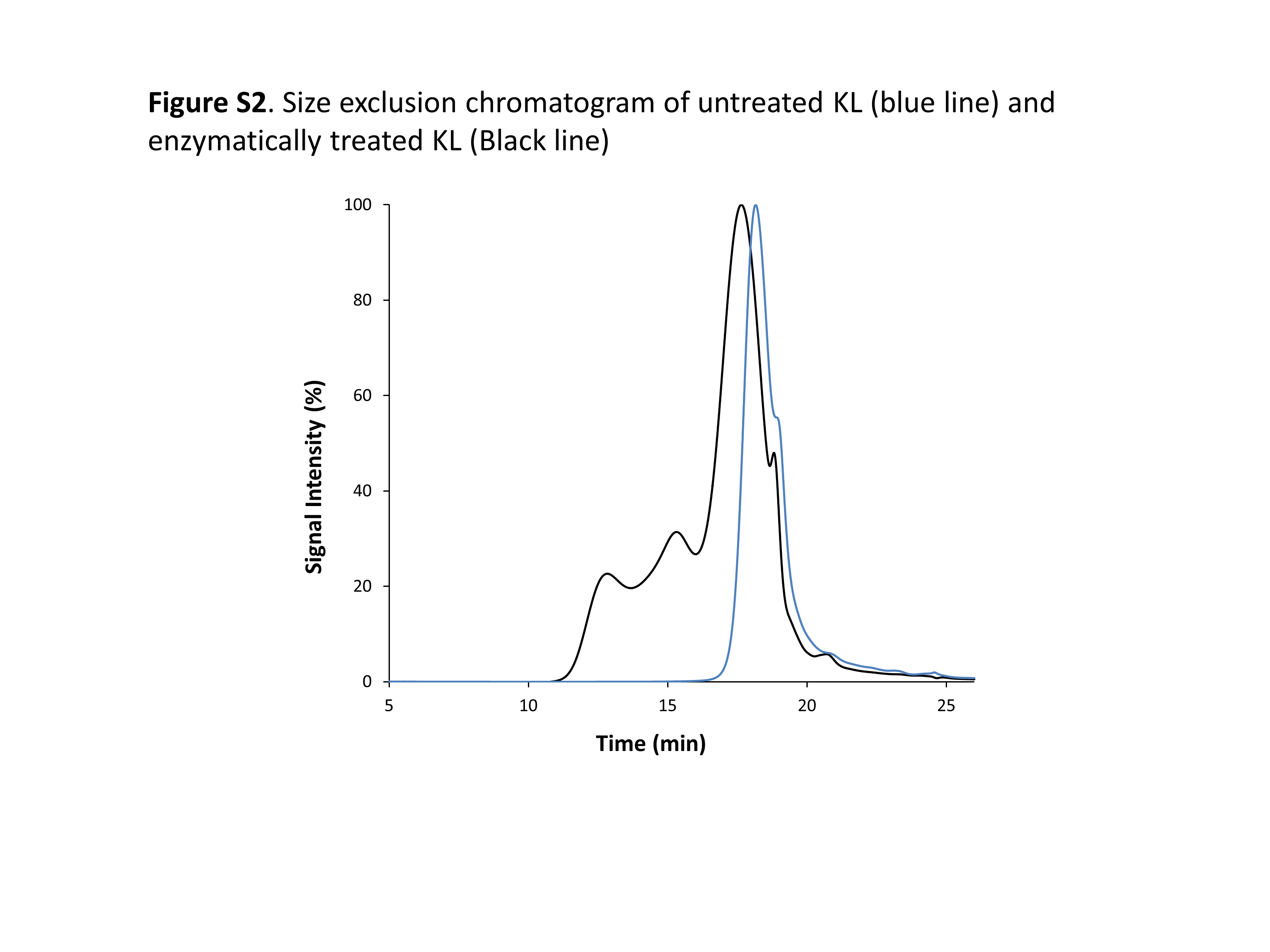

Supplement: Supplementary file 1 [file polymers-10-00642-s001.zip › Figure S2.TIF]

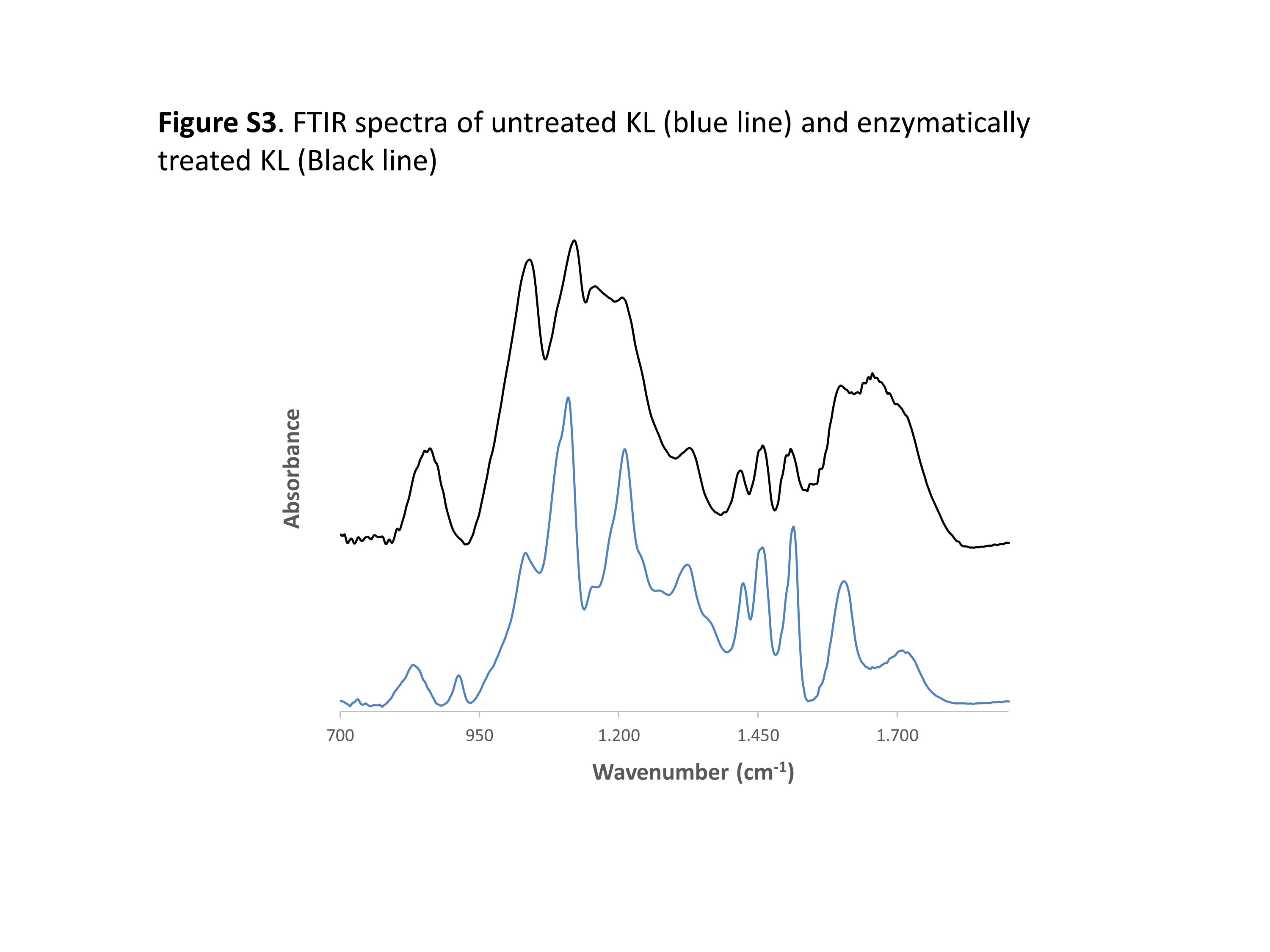

Supplement: Supplementary file 1 [file polymers-10-00642-s001.zip › Figure S3.TIF]
